# Health, function, quality of life and self-esteem in AIS; preliminary results from BrAIST

**DOI:** 10.1186/1748-7161-9-S1-O80

**Published:** 2014-12-04

**Authors:** Lori Dolan, Stuart Weinstein

**Affiliations:** 1 University of Iowa, Iowa City, IA, USA

## Background

Conflicting reports have been published concerning the effect of adolescent idiopathic scoliosis (AIS) and its treatment on patient physical and psychosocial function and overall quality of life.

## Aim

The aim of this study is to compare the physical and psychosocial function and quality of life over time in treated and untreated patients and in comparison to school-based populations.

## Methods

BrAIST, a multi-center, partially randomized prospective study, enrolled 383 subjects with AIS and followed them until skeletal maturity (treatment success) or until the curve exceeded 50 degrees (treatment failure). Patients were braced or observed based on randomization or on their own preference. They completed the Child Health Questionnaire (CHQ) (1) and the PedsQL (QOL) (2) prior to treatment and then every six months. Baseline and final follow-up scores were ranked and compared using analysis of variance techniques. Scores were also compared to published norms. (2, 3)

## Results

Baseline, final scores and final outcome were available for 237 subjects. 61% were braced. The success rate was 49% in the untreated group compared to 72% in the braced group. There were no statistically significant differences between the braced and untreated groups on any of the subscales at baseline. At final follow-up, there is some evidence that patients who were braced yet had treatment failure ranked lower than braced patients with a successful outcome, on the self-esteem (p<0.07), behavior (p<0.07), physical functioning (p<0.09) and role functioning (p<0.05) CHQ subscales. This difference was also found in QOL scores (p<0.04). Overall, subject QOL and CHQ scores were not different than those published for school-based populations.

## Conclusions

Patients in this study scored similar to those in a school-based population, indicating little effect of the diagnosis of AIS on their physical and psychosocial function or overall quality of life. However, there were differences within the study cohort. Developing a curve of 50 degrees or greater was possibly associated with lower-ranked self-esteem, behavior, physical and role functioning and overall QOL. These findings suggest that patients who wear a brace require not only orthopaedic support, but also psychosocial support, especially in the face of significant curve progression.

